# Rate-Dependent Effects of Black Soldier Fly Frass on Germination, Shoot Growth, and Nodulation of Forage Legumes

**DOI:** 10.3390/plants15091388

**Published:** 2026-04-30

**Authors:** Simon Hodge, Larisa-Georgiana Ciobanu, Brian Gormley

**Affiliations:** School of Agriculture & Food Science, University College Dublin, D04 V1W8 Dublin, Ireland; larisa.ciobanu1902@gmail.com (L.-G.C.); brian.gormley@grassland.ie (B.G.)

**Keywords:** circular economy, *Hermetia illucens*, clovers, *Rhizobium*, *Trifolium*

## Abstract

Insect frass fertilisers (IFFs) are increasingly promoted as sustainable soil amendments within circular agricultural systems. However, the compatibility of IFFs with nitrogen-fixing legumes is poorly understood. This study evaluated the effects of a fertiliser produced from *Hermetia illucens* frass (HexaFrass™; HF) on germination, seedling emergence, shoot growth, and root nodulation in six forage legume species (*Trifolium repens* L., *T. pratense* L., *T. incarnatum* L., *T. hybridum* L., *Melilotus albus* Medik., and *Medicago lupulina* L.). Aqueous HF extracts (1% *w*/*v*) had no significant effect on seed germination, whereas higher concentrations (10% *w*/*v*) reduced germination in both *T. pratense* and *T. incarnatum*. In glasshouse trials, incorporation of HF (3 g per pot) did not affect seedling emergence but significantly increased shoot biomass across all plant species tested, with growth responses comparable to, or exceeding, those obtained with an equivalent mass of organic chicken manure. Across species, the shoot dry weight of the HF-treated plants was over nine times that obtained in the unfertilised control plants. Plant responses to HF application rate were non-linear, with maximum shoot biomass achieved at intermediate doses (~4–5 g per pot). Root nodulation exhibited a similar dose-dependent pattern: low HF application rates slightly enhanced nodulation, whereas higher rates suppressed nodule numbers. These findings indicate that IFFs can promote early growth of forage legumes, but reinforce that for each plant system (plant species, growing conditions, growing medium etc) there is a need to optimise fertiliser application rates to balance nutrient supply while avoiding the inhibitory effects observed at high rates. Further work is needed to establish the compatibility of IFFs with forage legumes in long-term trials performed under field conditions.

## 1. Introduction

The industrial-scale production of insects for animal feed and human consumption has expanded considerably in recent years, resulting in the generation of substantial quantities of ‘frass’ as a residual byproduct [1,2,3,4]. This material, composed of insect faeces, shed exoskeletons, growing media, and associated microorganisms, is increasingly recognised as a valuable source of plant nutrients. Frass typically contains appreciable concentrations of nitrogen, phosphorus, and potassium, and can be processed through drying, heat treatment, or pelleting to produce commercial organically-derived fertilisers and soil amendments [5,6,7,8,9,10]. The valorisation of this waste stream aligns closely with circular bioeconomy principles, whereby organic residues are repurposed into value-added products, reducing total waste and enhancing resource-use efficiency [11,12,13].

To develop more sustainable operations, many farmers now aim to maintain soil fertility while reducing inputs of environmentally-damaging synthetic fertilisers. Consequently, insect frass fertilisers (IFFs) have attracted growing interest as sustainable alternatives to conventional fertilisers, particularly within low-input and regenerative agricultural systems [5,14,15,16,17]. Insect frass fertilisers have been shown to improve the growth and yield of several important vegetable, cereal, and pasture species [18,19,20,21]. Additionally, exposure to frass fertilisers or liquid extracts has been found to enhance plant resistance to a range of abiotic stresses [22,23,24,25], as well as pest species such as insects [26], nematodes [27], fungi [28,29], and bacteria [30]. In terms of pasture-based grazing systems, IFFs can increase growth of several important grass species, such as Perennial Ryegrass (*Lolium perenne* L.), Italian Ryegrass (*Lolium multiflorum* Lam.), Timothy (*Phleum pratense* L.), Cocksfoot (*Dactylis glomerata* L.), and Bermudagrass (*Cynodon dactylon* (L.) Pers.) [5,20,31,32,33,34,35,36]. Additionally, IFF produced from BSF frass increases the shoot and leaf growth of herbaceous forage plants (Chicory; *Cichorium intybus* L. and Plantain; *Plantago lanceolata* L.) frequently included in multi-species grazing swards [37].

The move away from monoculture grasslands towards multispecies pastures, particularly with the inclusion of forage legumes such as clover, is becoming increasingly widespread, as legumes offer both high quality forage and the ability to add nitrogen to soil via their symbiotic relationships with nitrogen-fixing bacteria [38,39]. The quantity of fixed atmospheric nitrogen can be substantial, with some estimates suggesting that ryegrass/white clover (*Lolium perenne*/*Trifolium repens* L) swards would produce the same dry matter as ryegrass monocultures receiving 200 kg N.ha^−1^.yr^−1^ [38].

Several legumes have shown positive responses to the application of frass fertilisers, for example: common bean [*Phaseolus vulgaris* L.) [40,41], soybean (*Glycine max* (L.) Merr.) [42,43,44], long bean (*Vigna unguiculata* (L.)) [45], and cluster bean (*Cyamopsis tetragonoloba* (L.) Taub.) [46]. Kidd et al. examined the effects of frass fertiliser on swards of red clover (*Trifolium pratense* L.) under field conditions, and reported a 1.5-times increase in shoot yield compared with a no-fertiliser treatment [33]. In general, however, the compatibility of insect frass fertilisers (IFFs) with nitrogen-fixing pasture legumes remains poorly understood. Similarly, the compatibility of applying IFFs with the maintenance, or promotion, of root–microorganism symbioses has not been extensively studied. IFFs have been shown to increase root nodulation of common bean (*Phaseolus vulgaris* L.) [40], whereas colonisation of tomato roots by arbuscular mycorrhizal fungi was inhibited at high application rates of an IFF [47].

To address some of these gaps in the scientific literature, this study aimed to provide preliminary data on the compatibility of IFFs and forage legumes. To enhance the generalisability of the results, six species of clover were included in our experiments, and we assessed multiple aspects of plant performance, ranging from establishment (germination and seedling emergence) to vegetative growth (shoot fresh weight and dry weight) and symbiotic interactions (root nodule formation). To meet these aims, a series of laboratory and glasshouse experiments were conducted to assess: (1) the effects of aqueous IFF extracts on seed germination; (2) the influence of IFF on seedling emergence; (3) the impact of different application rates of IFF on shoot growth; and (4) the effects of incorporating IFF into growing media on root nodulation.

## 2. Materials and Methods

### 2.1. General Methods

To examine the robustness of the results across plant species, six plant species were used in this study: white clover (WC; *Trifolium repens* L.), red clover (RC; *Trifolium pratense* L.), crimson clover (CC; *Trifolium incarnatum* L.), Alsike clover (AC; *Trifolium hybridum* L.), Bokhara clover (BC; *Melilotus albus* Medik.), and yellow clover (YC; *Medicago lupulina* L.). All seeds were obtained from Fruit Hill Farm, Co., Cork, Ireland. All of these species are used as forage crops, cover crops, or as N-fixing green manures. The latter two species are not true clovers but were included as they are closely related to clovers and are sold as N-fixing or pollinator-friendly cover crops in Ireland. *Medicago lupulina* is sometime referred to as ‘black medick’ or ‘hop clover’, whereas *Melilotus albus* is known as ‘honey clover’ or ‘sweet clover’ because high levels of coumarin cause it to be highly aromatic.

HexaFrass^TM^ (HF) is a commercial fertilizer marketed by HexaFly, Co., Meath, Ireland. HF is produced via rearing Black Soldier Fly (BSF; *Hermetia illucens*) larvae on brewery waste, and the final product typically contains 8% mineral ash and 60% organic matter (39% dietary fibre). HF is rich in chitin, and has an NPK ratio of approximately 4:2:1 with a protein content of 24%. Small amounts of fatty acids (0.2%) are also present. Nutrient analysis has identified additional micronutrients such as boron (8.7 mg/kg), zinc (93 mg/kg), sulphur (6 g/kg), magnesium (5 g/kg), iron (300 mg/kg), and copper (12 mg/kg). The pH of an aqueous 1:1 HF solution was found to be approximately neutral at 7.3 [19].

In order to compare the effects of HF with a standard fertilizer, we used another organically-certified fertilizer, Westland Organic Chicken Manure Pellets (CM; NPK of 4.5:3.5:2.5). This same fertiliser has been used in several previous studies as a positive control for comparison with plant performance resulting from application of HF [18,19,37,47].

### 2.2. The Effect of HexaFrass Extracts on Clover Seed Germination

For the germination trials, 25 seeds were arranged approximately 5 mm apart in a 5 × 5 grid on two filter papers (Whatman’s No 1) set inside plastic Petri dishes (9 cm diameter). The filter papers were dampened using 5 mL untreated water (0% HF) or with 5 mL of the appropriate HF extract. The edges of the Petri dishes were sealed using Parafilm, and the Petri dishes were placed in a dark cupboard at room temperature (approximately 25 °C). Germination was checked every 2 or 3 days for 14 days. The seeds were viewed under a low-power microscope and were classified as having germinated if roots or shoots over 1 mm in length had appeared. Any germinated seeds were removed from the Petri dish. After checking, additional water or HexaFrass extract was added to the filter papers if needed, and the Petri dishes were resealed with fresh Parafilm and then returned to the darkened cupboard.

#### 2.2.1. The Effect of Low Concentration HexaFrass Extract on Clover Seed Germination

To investigate the effect of aqueous HF extract on germination in all six plant species, a 1% *w*/*v* extract was produced by adding 10 g of powdered HF to 1 L of unmodified tap water. The mixture was stirred using a magnetic stirrer (no heat) overnight for approximately 18 h, and was then passed through a fine metal sieve. In each trial there were four replicate Petri dishes (100 seeds in total) for each clover species for both the control and the HF treatment.

#### 2.2.2. The Dose-Related Effect of HexaFrass on Clover Seed Germination

To examine the effect of HF extract concentration on germination, a 10% *w*/*v* extract was initially created by mixing 10 g of HF with 100 mL tap water, as described above. This extract was then serially diluted to produce the equivalent of 5%, 2.5%, and 1.25% *w*/*v* solutions. Germination trials were conducted as described above using CC and RC: these clover species were selected for further testing because they exhibited intermediate germination rates in the initial trial, and therefore would allow us to detect both positive and negative effects of the HF extracts on germination. There were four replicate Petri dishes (100 seeds in total) for the two clover species for each of the HF treatments and the water-treated controls.

### 2.3. Glasshouse Pot Trials

Seedling emergence and plant growth trials were performed in a glasshouse at Rosemount Environmental Research Station, University College Dublin (53.305349, −6.233302), between February and August 2024. During this period the glasshouse had an average daily temperature of approximately 18 °C (range 15–33 °C) and average daily relative humidity of 51% (range 42–83%). No artificial lights were used throughout the trials. In each trial, the experimental treatments were arranged on glasshouse benches using complete randomised designs.

All glasshouse trials were performed using 7 cm × 7 cm square plastic pots with a potting mix consisting of equal parts by volume of Westland Nutrient Rich Garden Soil, Plagron Coco Bric coir fibre, and vermiculite. A small amount (~200 g) of a rich loam topsoil from an outdoor horticultural area was mixed in with each batch of potting mix to increase microbial diversity and improve the chances of root nodulation. As the coir fibre and vermiculite contain negligible quantities of nutrients, this potting mix was considered a ‘low nutrient’ growing medium, with chemical analysis (Southern Science Laboratories, Kerry, Ireland) indicating N–P–K values of 0.3–0.02–0.5 by dry weight [19].

Fertiliser treatments were added to each pot individually and mixed in with the growing media prior to seeds being sown. The plants were watered with untreated water every 2 to 3 days, and were always watered the day prior to harvesting. During the experiments, the pots were placed on individual plastic saucers (10 cm diameter) to prevent leaching of nutrients and contamination across fertilizer treatments.

#### 2.3.1. The Effect of HexaFrass on Clover Seedling Emergence

To examine the effect of HF on emergence of clover seedlings, five clover seeds were sown (at approx. 5 mm depth) in pots after the addition of 3 g HF, 3 g CM, or no fertiliser. Five clover species were tested in this trial (AC, BC, CC, RC, WC) with eight replicate pots per clover species per fertiliser treatment (120 pots in total). Emergence of seedlings was monitored every 2–3 days for 22 days after sowing. A seedling was considered as ‘emerged’ when the shoot had broken the surface of the soil and seed leaves were visible.

#### 2.3.2. Comparison of HexaFrass and Chicken Manure Fertiliser on Performance of Clover Seedlings

To compare the effects of HF with those obtained when using a standard organic fertiliser, plants were grown in pots with the addition of 3 g HF, 3 g CM, or no fertiliser. Five clover species were tested in this trial (AC, BC, CC, RC, WC) with eight replicate pots per clover species per fertiliser treatment (120 pots in total). Initially, several clover seeds were sown per pot, and then thinned down to one seedling per pot after 14 days.

Plants were harvested 6 weeks after sowing. At harvest, stems and foliage (‘shoot’) were cut at the soil surface, and shoot fresh weight (Fwt) was obtained. All shoots were placed in paper bags, dried in an oven at 65 °C for 3 d, and then the dried shoot weight (Dwt) was obtained. Shoot dry matter content (DM; %) was calculated as 100 × (Dwt/Fwt).

After the plant shoots had been harvested, the roots of each plant were carefully removed from the potting media. The extent of root nodulation was scored on a scale between 0–5, with 0 indicating no nodules, 1 indicating one or a few small nodules, 2 indicating several small nodules, 3 indicating moderate/obvious nodulation, and 4 to 5 indicating abundant or extensive nodulation. Half scores were allowed if nodulation was thought to have fallen between two categories. Several previous studies have used similar scoring systems to classify plants in terms of the extent of root nodulation [48,49,50]. Additionally, the use of rapid visual assessment of root properties has been validated by demonstrating significant positive correlations with quantitative measurements such as nodule number and nodule dry weight [51].

#### 2.3.3. The Effect of HexaFrass Application Rate on Performance of Clover Seedlings

To assess the effect of HF application rate on clover performance, pots were set up with 0, 0.5, 1, 2, 4, 6, 8, and 10 g of dry HF per pot. This experiment used WC and yellow clover YC, with five replicates of each application rate per clover species, except for the 8 g and 10 g treatments, where only three replicates were used. Plants were harvested after 7 weeks and processed as described above.

### 2.4. Statistical Analysis

Data were collated and graphics created using Microsoft Excel. All statistical analyses were performed using Genstat v21 [VSN International Ltd., Hemel Hempstead, UK]. After model fitting, approximations to normality of errors and equality of variances were checked for compliance by visual inspection of residual plots [52].

For the germination trials investigating how seeds of six clover species responded to the 1% HF extract, the number of seeds germinated in each Petri dish (*x*/25) after 14 days was designated as a binomial response variable. A generalised linear model, using a logit link function, was then used to examine how germination was affected by clover species and HF treatment. Similarly, in the seedling emergence trial, the number of seedlings that had emerged in each pot (*x*/5) after 22 days was used as the binomial response variable. A generalised linear model, using a logit link function, was then used to examine how emergence was influenced by clover species and fertiliser treatment.

In the trials investigating how different clovers responded to HexaFrass and chicken manure, a separate one-way ANOVA was performed for each response variable (Fwt; Dwt; DM) for each of the five clover species. To look for overall trends, an additional analysis was performed for each response variable by pooling all data, and then assigning clover species as a random factor and fertiliser as a fixed factor in a mixed model. For each analysis, pairwise comparisons among the three fertiliser treatments were made using Fisher’s Least Significant Difference (LSD; *p* < 0.05). Nodule score data were analysed by a non-parametric Kruskal–Wallis test followed by pairwise Mann–Whitney tests.

In the examination of HF application rate (g per pot) on clover performance, and when examining the effect of HF extract concentration (%; *w*/*v*) on seed germination, polynomial regression curves were fitted using a quadratic model of the form:

*y*
= *a* + *b*(HF) + *c*(HF^2^).

where *a*, *b*, and *c* are constants and HF is the application rate or extract concentration. For these polynomial relationships, HF_Max_, which is the HF value that would produce the maximum predicted value of the response variable, was estimated using the formula −*b*/2*c*.

## 3. Results

### 3.1. The Effect of HexaFrass™ on Clover Seed Germination

**Figure 1:**
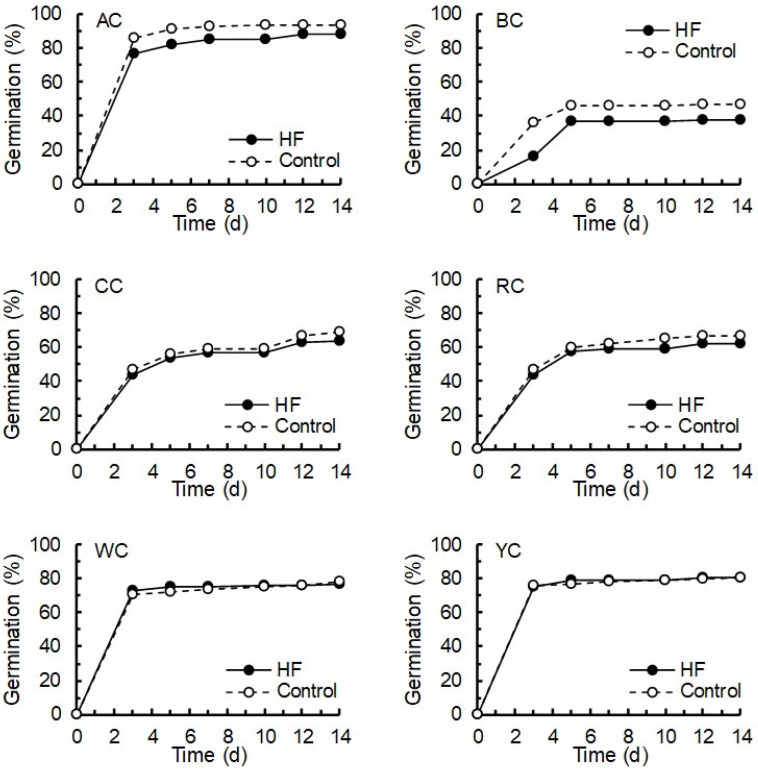
Germination of clover seeds over 14 days when treated with water or a 1% *w*/*v* HexaFrass extract. Seeds were maintained in Petri dishes at room temperature in dark conditions. The values given are a proportion (%) of 100 seeds (4 × 25 per Peri dish) in each treatment for each species. AC—Alsike clover; BC—Bokhara clover; CC—crimson clover; RC—red clover; WC—white clover; YC—yellow clover.

There were significant differences among the six clover species in terms of seed germination (χ^2^_5, 41_ = 27.89, *p* < 0.001), ranging from AC with a 14 day germination rate of 91% to BC with a final germination rate of 42% (Figure 1). For all six clover species, the trends in germination over time were similar for the seeds treated with 1% *w*/*v* HF extract and for the control water-treated seeds (Figure 1). When the data were pooled across all species, there was moderate evidence that HF-treated seeds had a slightly lower germination rate (68.3%) than the control seeds treated with water (72.7%) (χ^2^_1, 41_ = 3.06, *p* = 0.080), but even this small effect was not apparent for all clover species (e.g., WC and YC) (Figure 1).

**Figure 2:**
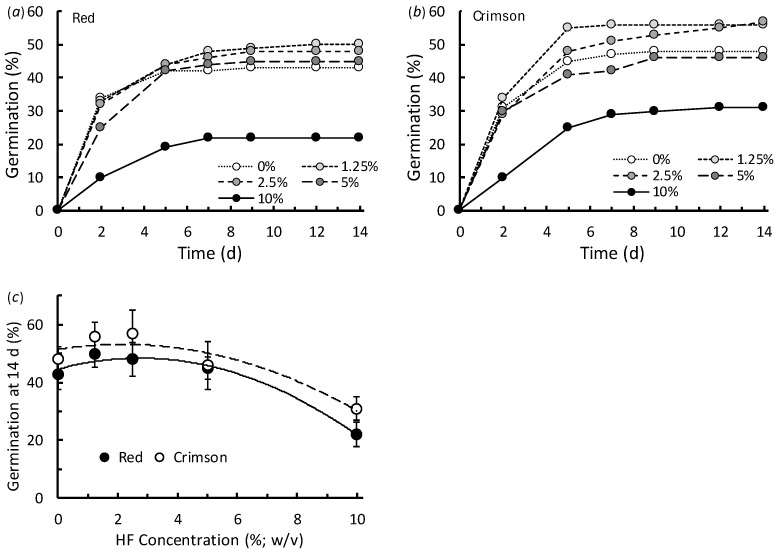
Germination (%) of (**a**) red and (**b**) crimson clover seeds in Petri dishes at room temperature over 14 days when treated with water or a range of HexaFrass extracts ranging from 1.25% to 10% *w*/*v*. Graph (**c**) shows the relationships between germination (%; mean ± SE) at 14 days with extract concentration. Fitted polynomial curves: CC germination = −0.37(HF^2^) + 1.62(HF) + 51.46, *r*^2^ = 0.88; RC germination = −0.51(HF^2^) + 2.78(HF) + 44.62, *r*^2^ = 0.98.

When investigating the effects of HF extract concentration on germination, there was no significant difference in average germination rates between RC seeds and CC seeds (χ^2^_1, 30_ = 2.76, *p* = 0.107; Figure 2). There were, however, clear differences in germination rates among the different HF treatments (χ^2^_4, 30_ = 7.35, *p* < 0.001; Figure 2). The relationships between germination and HF concentration were similar for both clover species, in that the seeds treated with the strongest (10% *w*/*v*) HF extract had much reduced germination compared with the other treatments (Figure 2). The polynomial models fitted to germination at 14 days indicated that although the 10% HF inhibited germination, exposure of seeds to a low concentration of HF extract may have had a slight positive effect. The polynomial models suggested that, for CC, the predicted maximum germination rate was 53.3% with a HF concentration of 2.2% *w*/*v*, and for RC the maximum predicted germination was 48.3% with a HF concentration of 2.7% *w*/*v* (Figure 2c).

### 3.2. The Effect of HexaFrass™ and Chicken Manure on Clover Seedling Emergence

**Figure 3:**
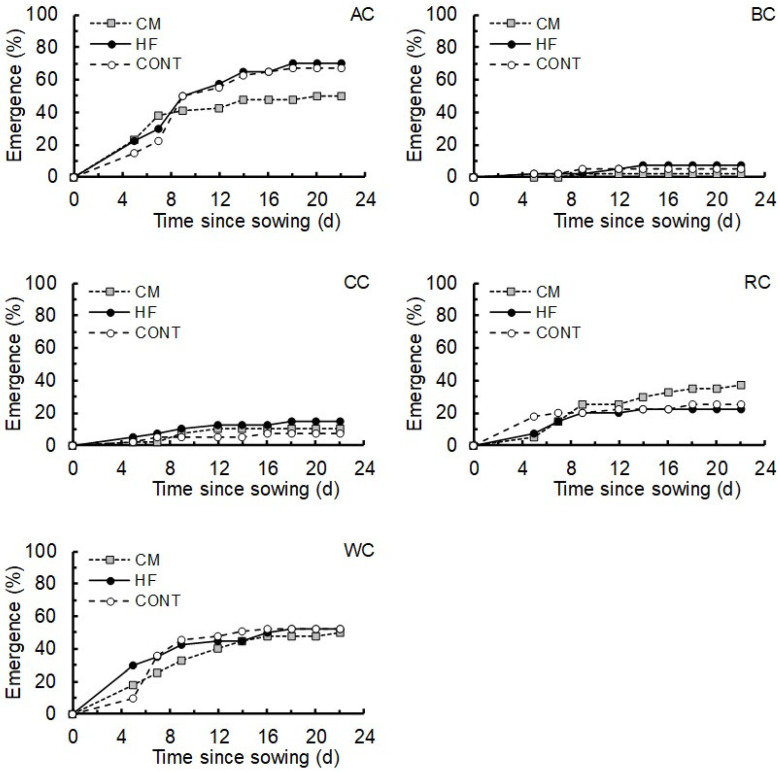
Emergence of clover seedlings over 22 days when sown in pots containing 3 g HexaFrass (HF), 3 g chicken manure (CM), and no-fertiliser controls (CONT). The values given are a proportion (%) of 80 seeds in each treatment for each species. AC—Alsike clover; BC—Bokhara clover; CC—crimson clover; RC—red clover; WC—White clover.

There were clear differences in the seedling emergence rates of the different clover species (χ^2^_4, 105_ = 25.80, *p* < 0.001), with BC (5%) and CC (11%) having particularly low emergence rates (Figure 3). The patterns in seedling emergence over time were very similar for the control and HF treated plants in all five clover species tested (Figure 3). Although there was some variability in the effects of CM on emergence of different clover species, overall there was no significant effect of fertilizer treatment (χ^2^_2, 105_ = 0.20, *p* = 0.822), or interaction between clover species and fertilizer treatment (χ^2^_8, 105_ = 0.62, *p* = 0.756) on seedling emergence rate as determined at 22 days (Figure 3).

### 3.3. Shoot Growth Response of Five Clover Species to Addition of HexaFrass™ and Chicken Manure

In the trials comparing HF with chicken manure (CM), HF increased shoot Fwt and Dwt compared with the no-fertiliser control treatment for all five clover species tested. HF also produced an equivalent, or greater, shoot Fwt and Dwt than that obtained with the CM (Table 1). Over all plants, HF caused a higher (nine-fold) increase in shoot Dwt compared with CM (6.5-fold).

Shoot DM% was reduced in all clover species with the addition of either HF or CM, but this effect was not statistically significant for BC (Table 1). Taken over all five species, both HF and CM reduced shoot DM% by around 33% (Table 1).

### 3.4. Response of Yellow Clover and White Clover Shoot Growth to HexaFrass™ Application Rate

**Figure 4:**
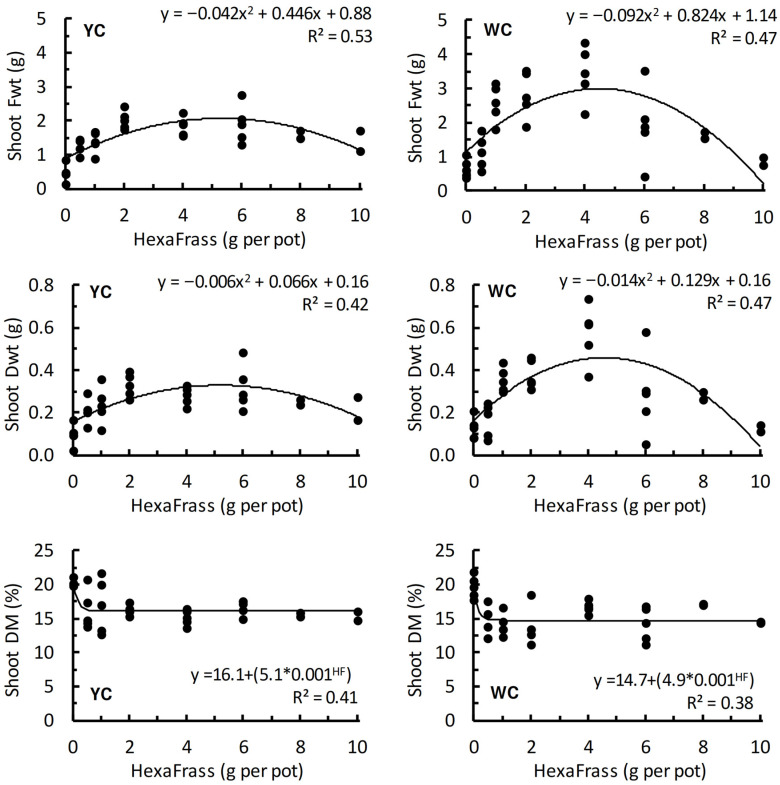
Shoot fresh weight (Fwt; g), dry weight (Dwt; g), and dry matter content (DM; %) of yellow clover (YC) and white clover (WC) in response to different application rates of HexaFrass fertiliser (g per pot).

Shoot Fwt, Dwt, and DM in both yellow (YC) and white clover (WC) exhibited non-linear responses to HF application rate (Figure 4). Trends for shoot Fwt and Dwt could be described by quadratic curves within the range of HF application rates tested, whereas shoot dry matter content (%) followed asymptotic trends (Figure 4).

For both YC and WC, increases in shoot weight (compared with the no HF control treatments) were observed at relatively low application rates of around 1 g HF per pot. For YC, shoot weight peaked at around 5 g HF per pot. Similarly, for WC the peak in shoot weight occurred at around 4.5 g HF per pot (Figure 4).

Shoot DM (%) was negatively affected by the addition of HF, and decreases in DM were apparent in both clover species at low HF application rates. At application rates of approximately ≥2 g HF per pot the shoot DM content of both clover species levelled off, at 16% for YC and 14.7% for WC (Figure 4).

**Table 1:** The response of five clover species to the addition of HexaFrass (HF; 3 g/pot) or chicken manure fertiliser (CM; 3 g/pot) in a glasshouse trial. Controls had no fertiliser treatment. Values given are mean ± SE (*n* = 8). Within each row, values with different letter codes indicate significant difference, as indicated by Fisher’s LSD *p* < 0.05. F and *p* values calculated using one-way ANOVA. * Nodule score data were analysed by non-parametric Kruskal–Wallis tests followed pairwise Mann–Whitney tests.

Response	Clover	Control	HF	CM	LSD	F	*p*
Fwt (g)	Alsike	0.13 ± 0.06 ^a^	1.75 ± 0.10 ^b^	1.49 ± 0.20 ^b^	0.38	44.4	<0.001
	Bokhara	0.18 ± 0.03 ^a^	2.26 ± 0.17 ^b^	1.83 ± 0.25 ^b^	0.52	39.3	<0.001
	Crimson	0.24 ± 0.05 ^a^	3.19 ± 0.15 ^c^	2.13 ± 0.30 ^b^	0.58	57.6	<0.001
	Red	0.36 ± 0.05 ^a^	2.49 ± 0.37 ^c^	1.39 ± 0.27 ^b^	0.78	16.2	<0.001
	White	0.08 ± 0.02 ^a^	1.44 ± 0.13 ^b^	1.06 ± 0.19 ^b^	0.39	17.6	<0.001
	**ALL**	0.20 ± 0.02 ^a^	2.22 ± 0.13 ^c^	1.58 ± 0.12 ^b^	0.25	132.4	<0.001
Dwt (g)	Alsike	0.031 ± 0.011 ^a^	0.314 ± 0.019 ^b^	0.260 ± 0.036 ^b^	0.071	38.4	<0.001
	Bokhara	0.037 ± 0.007 ^a^	0.426 ± 0.035 ^b^	0.354 ± 0.046 ^b^	0.098	38.4	<0.001
	Crimson	0.051 ± 0.009 ^a^	0.518 ± 0.043 ^c^	0.324 ± 0.046 ^b^	0.107	41.2	<0.001
	Red	0.059 ± 0.007 ^a^	0.362 ± 0.056 ^c^	0.186 ± 0.029 ^b^	0.107	17.3	<0.001
	White	0.018 ± 0.003 ^a^	0.211 ± 0.023 ^c^	0.145 ± 0.024 ^b^	0.057	25.9	<0.001
	**ALL**	0.039 ± 0.004 ^a^	0.366 ± 0.023 ^c^	0.254 ± 0.021 ^b^	0.042	119.9	<0.001
DM (%)	Alsike	42.5 ± 8.7 ^a^	18.0 ± 0.8 ^b^	17.4 ± 0.5 ^b^	14.8	8.1	0.002
	Bokhara	19.6 ± 1.1	18.8 ± 0.5	19.5 ± 0.6	2.3	0.3	0.735
	Crimson	22.7 ± 2.0 ^a^	16.2 ± 0.9 ^b^	15.1 ± 0.4 ^b^	3.8	10.1	<0.001
	Red	16.9 ± 0.8 ^a^	14.7 ± 0.8 ^ab^	13.9 ± 0.8 ^b^	2.4	3.6	0.045
	White	23.1 ± 1.6 ^a^	16.2 ± 2.8 ^b^	14.9 ± 1.4 ^b^	5.9	4.8	0.019
	**ALL**	25.0 ± 2.3 ^a^	16.8 ± 0.6 ^b^	16.2 ± 0.5 ^b^	3.6	14.7	<0.001
Nodules *	Alsike	0.9 ± 0.4	1.1 ± 0.2	1.0 ± 0.2	-	1.27	0.531
	Bokhara	0.0	0.0	0.0	-	-	-
	Crimson	1.0 ± 0.3	1.5 ± 0.2	1.9 ± 0.2	-	5.43	0.066
	Red	1.1 ± 0.2	1.5 ± 0.3	1.9 ± 0.3	-	3.39	0.184
	White	0.1 ± 0.1 ^a^	1.3 ± 0.2 ^b^	1.1 ± 0.4 ^b^	-	9.69	0.008
	**ALL**	0.6 ± 0.1 ^a^	1.1 ± 0.1 ^b^	1.2 ± 0.2 ^b^	-	14.00	<0.001

### 3.5. The Effect of Frass Fertiliser on Nodulation of Clover Roots

In the trial examining the effect of HF (3 g per pot) on nodulation in five clover species (see Section 2.3 and Section 3.3), the nodule scores (0–5) were mainly low. From the 160 plants scored for nodules, 60 had no nodules, and an additional 58 plants were assigned a nodule score of 1. Seven of the plants were given scores of three, indicating that roots were well nodulated, but no plants were given nodule scores of four or five. Over all five clover species tested, the average nodule score for the HF-treated plants was 80% higher than that achieved in the control plants (Table 1). No nodules were observed on the roots of any BC plants; for the other four clover species the average nodule scores were higher for HF-treated than for the control plants, however this difference was statistically significant only for WC (Table 1).

**Figure 5:**
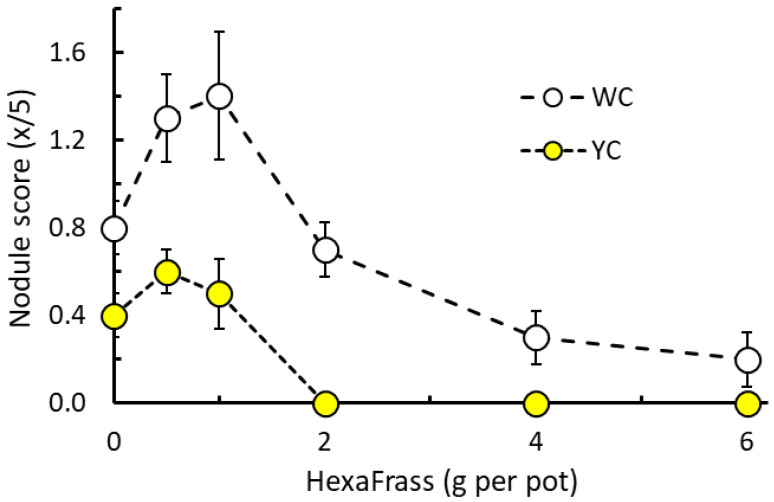
Root nodule score (*x*/5) of white clover (WC) and yellow clover YC) in response to differing application rates of HexaFrass fertiliser (g per pot). Values given are means ± SE.

In the trial investigating HF application rate (0–6 g per pot) on clover performance (see Section 2.4 and Section 3.4) it was found that nodule score for both white clover (*H*_5_ = 19.4, *p* = 0.002) and yellow clover (*H*_5_ = 22.0, *p* < 0.001) was significantly affected by the quantity of HF applied. The patterns for nodulation were similar for both YC and WC, where low application rates (0.5 g and 1 g HF per pot) appeared to induce a slight increase in nodulation, whereas at the higher application rates (≥4 g HF per pot) nodulation was reduced (Figure 5). The stimulation of nodulation at low HF application rates was most apparent for WC, whereas the inhibition of nodulation at high application rates was most apparent for YC.

## 4. Discussion

In general, the inclusion of moderate quantities of HexaFrass (HF) in growing media had a positive effect on shoot growth in all the clover species we tested. These findings are similar to several previous studies involving legume species, such as beans [40,41,42,45,46] and are supported by additional glasshouse trials we performed where the application of HF increased shoot growth of several agriculturally-important legume species (such as peas, vetch, alfalfa, lentils; see Appendix A, Table A1). Additionally, we found that the effect of incorporating HF in the growing media produced a plant growth response of similar magnitude to that achieved with a comparable organic fertiliser, dried chicken manure (CM), a finding which is supported by previous research [5,18,20,37,47]. For simplicity, we used equal weights of the two fertilisers as the basis for our comparison rather than attempt to balance nutrient application rates, but given that HF has slightly lower NPK values (4:2:1) than the CM (4.5:3.5:2.5) this does not detract from the comparable effects HF has on plant shoot growth.

When applying HF at different rates, there was an increase in shoot growth at low to medium application rates compared with the no-fertiliser controls, but at high rates the growth response was weaker. This non-linear response between shoot growth and IFF application rate has been observed in several previous studies [47,53,54,55], with seedling mortality being observed in chicory, plantain, and cereals [19,37] if HF was applied at high rates. Concentrations of soluble macronutrients in IFFs tend to be low, so it seems unlikely that these restraints on shoot growth are due to excessive soil N or P [19]. It is possible that the seedlings are being affected by other bioactive substances in the frass, such as chitin, but we did not test this explicitly and the precise mechanism behind the inhibition of plants exposed to high volumes of rates of IFFs still requires clarification.

Based on our results, it appears that treating plants with low application rates of dry HF or the application of dilute aqueous HF extracts would not majorly impact clover seed germination, seedling emergence, and plant establishment. Low concentrations of aqueous HF extracts did not inhibit germination, but RC and CC exhibited reduced germinations if they soaked in a more concentrated HF extract (10% *w*/*v*). Our results are similar to recent reports where frass extracts have inhibited seed germination in several plant species (e.g., lettuce, sunflower, cress, tomato, cabbages, kale), with germination often exhibiting a negative dose response relationship with the extract concentration [7,21,56,57,58,59]. Several recent studies have reported that applying IFFs to the soil surface and/or at high rates can have negative effects on germination and/or seedling emergence [35,60,61]. In this study, applying 3 g of dry HF to pots had no effect on seedling emergence, and further work is needed to see if the emergence of clovers (and other plants) is affected if higher rates are used.

The causal mechanism underlying IFF inhibition of germination is not yet fully understood. IFFs are not particularly high in macronutrients, and many of the more complex molecules present, such as chitin, are not readily water-soluble. Because frass fertilisers are typically not fully composted, germination inhibition may instead be related to high electrical conductivity, arising from elevated salt concentrations or specific ions such as Na, Ca, and NO_3_^−^ [7,15,58]. Kaczor [62] reported that BSF frass can also contain high levels of plant hormones, including salicylic acid, abscisic acid, indole-3-acetic acid, as well as cytokinins and auxins, all of which are involved in plant development and germination. At present, however, the mechanism responsible for the observed inhibition remains uncertain, and targeted studies are required to determine whether any of these chemical factors play a significant role.

Taken together, our results suggest that clover plants treated with low application rates of HF exhibited slightly higher nodulation than untreated plants, whereas high application rates appeared to inhibit nodulation. The stimulatory effects of organically derived fertilisers on nodulation have long been recognised and are typically attributed to improvements in organic matter, soil structure, aeration, microbial biomass, and overall soil health [63,64,65]. In addition, IFFs are known to influence the soil microbiome, including the diversity and relative abundance of nitrogen-fixing bacteria [66,67]. Chitin, a major component of IFFs, and its derivatives are also involved in molecular mechanisms of nodule development, and exogenous chitin has been shown to stimulate nodule formation [68,69].

Together, these factors suggest multiple direct and indirect pathways through which IFFs or their extracts may influence nodulation in legumes. However, there is limited research explicitly examining the effects of IFFs on root–microorganism symbioses. Chepkorir et al. reported that bush bean (*Phaseolus vulgaris* L.) treated with Black Soldier Fly frass exhibited significantly higher nodulation than both control plants and those treated with mineral NPK fertiliser [40]. In contrast, our results showed variable effects of HF on clover nodulation, and supplementary trials on *P. vulgaris* and other legumes (including peas, vetch, alfalfa, and lentils) similarly produced inconsistent nodulation responses to HF application (see Appendix A, Table A2). Given the relatively low levels of nodulation observed in our study, any conclusions regarding the effects of IFFs on nodulation and subsequent nitrogen fixation should be considered preliminary. Additionally, there were several experimental differences between our study and that of Chepkorir et al. [40] where a positive effect of IFF on nodulation and N-fixation was observed. For example, Chepkorir et al. [40] used a rhizobial inoculant, the IFF was created ‘in house’ and had been composted prior to being used, and the experiment was performed under field conditions over two growing seasons. Across all our experiments investigating nodulation, levels were generally low, and no nodules were observed on the roots of Bokhara clover. The outdoor topsoil incorporated into the growing medium was air-dried, sieved, and thoroughly mixed, and we assumed that variability among replicate pots would be minimal. However, it is possible that this soil lacked the appropriate rhizobia required to support nodulation in the clover species tested, and future studies would benefit from using commercially available inoculum to mitigate for this shortfall (although see [40]). A primary reason for including forage legumes in multi-species swards is N-fixation, and these inconsistencies between studies indicate that much further research is required to determine the range of conditions and time frames under which IFFs influence root nodulation.

We acknowledge there are several limitations associated with short-term glasshouse experiments such as those reported here. Plants are grown in small containers under controlled conditions that are largely free from pests and pathogens, and are harvested at an early developmental stage prior to flowering and seed production. However, such studies enable comparisons across multiple plant species and allow assessment of multiple key parameters relevant to forage performance, including germination, emergence, shoot growth, and nodulation [34,37]. Consistent with some previous studies on insect frass fertilisers, we observed variability in the effects among different experiments [20,47]. For instance, a 1% HF extract slightly reduced clover germination in one experiment, whereas a 1.25% extract produced a slight increase in germination in a second trial. Similarly, the mean nodulation score of WC increased with HF application in one experiment but decreased slightly in another. These findings highlight the importance of assessing the repeatability of plant responses to IFFs across multiple trials, using multiple plant taxa, and ideally under varying environmental and soil conditions, to establish robustness of results and distinguish consistently observed effects from those requiring further investigation.

## 5. Conclusions

As far as can be ascertained, this paper reports the first experiments investigating the effects of applying different rates of an IFF on multiple performance parameters of forage legume species. Overall, the results indicate that low to moderate application rates of HF, a commercial insect frass fertilizer derived from Black Soldier Fly frass, can enhance the growth of several clover species. Our results also highlight the importance of careful IFF dose management and establishing a situation-specific optimal application rate, as at high application rates IFFs can inhibit germination, reduce plant establishment, reduce levels of root nodulation, and reduce shoot growth. We accept that the presented results are preliminary, and caution is needed before extrapolating these results to a full-scale agricultural setting. Nevertheless, together with previous studies reporting positive effects of IFFs on pasture grasses and forage herbs, these new findings confirm that IFFs have potential as organically-derived soil amendments for low-input grazing systems, and that future research is warranted to evaluate IFFs on multispecies swards under field conditions.

## Data Availability

The original contributions presented in this study are included in the article. Further inquiries can be directed to the corresponding author.

## Appendix A

Summary of trials investigating the effects of HexaFrass fertiliser on shoot growth and nodulation in eight species of legumes in short term (6–8 week) glasshouse pot trials. Trials were performed under similar conditions as those described in the main paper. Nodule scores ranged from 0–5 as described in main text. Values given are mean ± SE. N—replications per treatment. *p*-value was obtained from a Mann–Whitney test.

**Table A1:** Shoot dry weight (g).

Common Name	Latin Name	N	0 g HF	3 g HF	*p*
Alfalfa	*Medicago sativa*	8	0.116 ± 0.016	0.421 ± 0.039	<0.001
Sanfoin	*Onobrychis viciifolia*	8	0.080 ± 0.012	0.217 ± 0.023	<0.001
Pinto bean	*Phaseolus vulgaris*	9	1.220 ± 0.130	1.630 ± 0.151	0.065
Pea	*Pisum sativum*	8	0.753 ± 0.074	1.168 ± 0.045	0.001
Vetch	*Vicia* sp.	8	0.281 ± 0.031	0.788 ± 0.028	<0.001
Tic bean	*Vicia faba*	8	1.421 ± 0.144	1.211 ± 0.195	0.593
Hairy Vetch	*Vicia hirsuta*	8	0.261 ± 0.013	0.735 ± 0.083	<0.001
Lentils	*Vicia lens*	10	0.666 ± 0.076	0.452 ± 0.043	0.023

**Table A2:** Root nodule scores (0–5).

Common Name	Latin Name	N	0 g HF	3 g HF	*p*
Alfalfa	*Medicago sativa*	8	0.00	0.00	-
Sanfoin	*Onobrychis viciifolia*	8	0.06	0.13	1.000
Pinto bean	*Phaseolus vulgaris*	9	0.22	0.28	1.000
Pea	*Pisum sativum*	8	1.19	1.50	0.155
Vetch	*Vicia* sp.	8	0.81	1.06	0.287
Tic bean	*Vicia faba*	8	1.25	0.50	0.076
Hairy Vetch	*Vicia hirsuta*	8	0.75	1.56	0.004
Lentils	*Vicia lens*	10	1.60	1.05	0.023
